# Effects of Adolescent Childbearing on Maternal Depression and Problem Behaviors: A Prospective, Population-Based Study Using Risk-Set Propensity Scores

**DOI:** 10.1371/journal.pone.0155641

**Published:** 2016-05-13

**Authors:** Alison E. Hipwell, Joseph Murray, Shuangyan Xiong, Stephanie D. Stepp, Kate E. Keenan

**Affiliations:** 1 Western Psychiatric Institute & Clinic, University of Pittsburgh Medical Center, Pittsburgh, Pennsylvania, United States of America; 2 Department of Psychology, University of Pittsburgh, Pittsburgh, Pennsylvania, United States of America; 3 Department of Psychiatry, University of Cambridge, Cambridgeshire, United Kingdom; 4 Postgraduate Programme in Epidemiology, Federal University of Pelotas, Pelotas, RS, Brazil; 5 Department of Statistics, University of Pittsburgh, Pittsburgh, Pennsylvania, United States of America; 6 Department of Psychiatry and Behavioral Neuroscience, University of Chicago, Chicago, Illinois, United States of America; St Francis Hospital, UNITED STATES

## Abstract

Adolescent mothers are reportedly at risk for depression and problem behaviors in the postpartum period, but studies have rarely considered developmental context and have yet to disentangle the effects of childbearing on adolescent functioning from selection effects that are associated with early pregnancy. The current study examined changes in adolescent depression, conduct problems and substance use (alcohol, tobacco and marijuana) across the peripartum period using risk-set propensity scores derived from a population-based, prospective study that began in childhood (the Pittsburgh Girls Study, PGS). Each of 147 childbearing adolescents (ages 12–19) was matched with two same-age, non-childbearing adolescents (n = 294) on pregnancy propensity using 15 time-varying risk variables derived from sociodemographic, psychopathology, substance use, family, peer and neighborhood domains assessed in the PGS wave prior to each pregnancy (T1). Postpartum depression and problem behaviors were assessed within the first 6 months following delivery (T2); data gathered from the non-childbearing adolescent controls spanned the same interval. Within the childbearing group, conduct problems and marijuana use reduced from T1 to T2, but depression severity and frequency of alcohol or tobacco use showed no change. When change was compared across the matched groups, conduct problems showed a greater reduction among childbearing adolescents. Relative to non-childbearing adolescents who reported more frequent substance use with time, childbearing adolescents reported no change in alcohol use and less frequent use of marijuana across the peripartum period. There were no group differences in patterns of change for depression severity and tobacco use. The results do not support the notion that adolescent childbearing represents a period of heightened risk for depression or problem behaviors.

## Introduction

Adolescent mothers face a variety of psychological, physical and economic challenges in the months following delivery [1–5], each of which has the potential to exacerbate stress and increase risk for postpartum depression [6–9]. For many adolescents, childbearing is also preceded by symptoms of depression [10, 11] and problem behaviors (e.g., conduct disorder and substance use) [12–17]. The convergence of these new and pre-existing risks suggests that the postpartum period is likely to be an important window of vulnerability for childbearing adolescents. Such a hypothesis, however, has yet to be tested using a rigorous prospective design.

A number of studies have shown that postpartum adolescents show elevated rates of depression, with some reports as high as 56% [3, 18–21]. This compares with rates of 10–15% depression prevalence among postpartum adults [7, 22], and rates below 10% among non-childbearing female adolescents [23]. However, results have been inconsistent and several studies have shown no association between adolescent childbearing status and severity of depression or emotional distress [24–27]. To a large extent these divergent findings reflect differences in sampling, measurement, and definitions of impairment. However, these mixed results may also reflect a lack of attention to developmental effects (e.g. changes in mood that typically occur with increasing age) or to early pregnancy selection effects that are likely to be confounded with experiences in the postpartum period. Both of these effects are highly relevant for understanding the relationship between childbearing and postpartum adjustment during adolescence.

In adult samples, approximately 50% of postpartum depressive episodes are a recurrence or exacerbation of a pre-existing disorder that has an onset prior to pregnancy [28, 29]. It is not known whether similar rates of depression continuity exist among adolescents in the transition to motherhood because adolescence is a developmental period that is characterized by considerable within-person variability in mood [30], and a time when rates of depressive disorder increase with age, especially among females [31–33]. Thus, in order to determine whether childbearing increases adolescent risk for depression in the postpartum period, we need to know the extent to which depressive symptoms are present prior childbearing, and the extent to which symptoms change relative to the normative changes of adolescence.

As far as we are aware, no prospective research has examined change in adolescent depression from pre-conception to the postpartum period. However, two longitudinal studies have assessed adolescent depression/distress from pregnancy to the postpartum period in childbearing adolescents as well as non-childbearing controls followed across a similar interval. In the first study, symptoms of depression reported by pregnant adolescents recruited from health clinics and educational programs were compared with those of non-childbearing acquaintance-controls of similar age [26]. The results revealed a significant reduction in depression severity from pregnancy to 6 weeks postpartum, but no differences between the childbearing and comparison groups in depression severity at any of the assessments, or in the extent of change in depression from pregnancy to 6 weeks postpartum. In the second study, the course of emotional distress was compared between pregnant and/or parenting adolescents with never-pregnant female adolescents recruited as part of an HIV/STD-risk study [27]. Again, a general reduction in distress across the study period was observed for all participants. However in this study the childbearing group reported significantly less emotional distress at four months postpartum compared with the non-childbearing adolescents. Although these studies were not able to address issues of selection due to the recruitment of already-pregnant or parenting adolescents who were also health clinic attendees, the results are important in suggesting that adolescent childbearing status does not appear to confer risk for depression when the effects of normative change are also taken into account.

Adolescence is a developmental period that is often associated with problem behaviors. In particular, alcohol, tobacco and marijuana use show steady increases across the adolescent years [34], whereas conduct problems tend to escalate, peak in mid-adolescence and then decline [35, 36]. Evidence is consistent in showing that adolescents are at elevated risk of using substances during pregnancy [37] with estimates ranging from 11% to 52% [38, 39]. Although longitudinal data indicate that the frequency of adolescent substance use tends to decline across pregnancy, use often resumes or rapidly increases again following delivery [39–41]. The extent to which the postpartum period represents a period of heightened risk for adolescent substance use however, remains unclear.

In comparison to substance use, there has been little prospective research examining change in adolescent conduct problems across the transition to motherhood. Some limited data suggest that conduct problems may decline simply because the maternal role limits opportunities to engage in antisocial behavior outside the home [42] and/or limits contacts with antisocial peers [43]. Other data also suggest that for a proportion of adolescents, childbearing represents a positive life choice [44, 45] that is associated with improved general functioning and reduced engagement in problem behaviors [46]. Nevertheless, little is known about the effects of childbearing on conduct problems relative to normative developmental change.

One of the major limitations of extant research examining adolescent postpartum adjustment stems from the strategy of sampling of adolescents who are already pregnant or parenting. Because adolescent childbearing, depression, conduct problems and substance use share many of the same background risk factors, it is possible that early childbearing does not play a causal role in eliciting postpartum depression or problem behaviors, but is instead a marker of some pre-existing vulnerability [27]. By sampling already-pregnant adolescents, the effects of childbearing on postpartum adjustment cannot be disentangled from the confounding effects of pre-existing risk factors that are associated with early pregnancy. Other factors that are consistently linked to early childbearing include social adversity, minority race, dysfunctional parenting experiences, sexual abuse, negative peer relations (e.g. peer victimization and deviant peer affiliation), and neighborhood problems [47–49]. These factors also serve as risk factors for the onset and course of depression, conduct problems and substance use [50, 51]. As a result, prospective research is needed that begins prior to pregnancy and addresses the problem of shared risks.

In the current study, a risk-set propensity score approach was used to parse out risk for postpartum depression and problem behaviors conferred by adolescent pregnancy selection factors from the putative effects of childbearing. Specifically, we examined changes in the severity of depression and conduct problems, and the frequency of tobacco, alcohol and marijuana use from pre-pregnancy (T1) to the postpartum period (T2) among childbearing adolescents, using data gathered as part of a large-scale, longitudinal, population-based study. We then examined these changes relative to those in non-childbearing adolescents matched at time T1 on their propensity to become pregnant in adolescence, and also matched on the duration of the interval from T1-T2.

Based on the results of prospective studies beginning in pregnancy, we first hypothesized that within the childbearing group, there would be an overall reduction in depression severity from pre-pregnancy to the postpartum period. We also expected that the severity of conduct problems among childbearing adolescents would decline during this period. In contrast, we expected to observe a mean increase in substance use frequency from pre-pregnancy to postpartum based on consistent evidence that substance use increases steadily across adolescence, and is likely to rebound or increase following delivery.

In our second set of hypotheses comparing childbearing with non-childbearing adolescents, we expected that decreasing levels of depression severity and conduct problems reported by the childbearing adolescents would contrast with developmental increases reported by the matched control group, giving rise to significant between-person differences in change over time. We also expected significant group differences for change in frequency of substance use. Thus, although we hypothesized that both childbearing and non-childbearing adolescents would report greater use of alcohol, tobacco and marijuana with increasing age, we expected the increase to be smaller among adolescents who transition to motherhood.

## Materials and Methods

### Sample and Procedures

The sample comprised adolescent participants of the Pittsburgh Girls Study (PGS); a longitudinal, population-based study of girls recruited following enumeration of 103,238 households in the city of Pittsburgh [52, 53]. In the enumeration process, every household in low-income neighborhoods (using 1990 census data on poverty), and a random sample of 50% of households in all other neighborhoods were contacted to identify an age-eligible girl (5–8 years old). Of 2,876 girls identified at the start of the study, 2,450 girls and their caregivers agreed to participate. Approximately half of the sample was African American (52%), 41% were European American, and the remaining girls were described as multiracial or representing another race. In wave 1, more than half of the caregivers were cohabiting and 39% of households received public assistance such as WIC, food stamps and Medicaid.

Participants in the current analyses were identified as part of the annual PGS interviews conducted between wave 5 (girls’ ages 9–12 years) and wave 13 (ages 17–20 years). In PGS wave 13, 2,109 participants (86.1% of the original sample) were retained in the study (mean retention across waves was 90.4%). From age 11, all PGS participants were asked annually whether they were currently pregnant, whether they were planning to carry the baby to term, and whether they had recently delivered a live baby. By wave 13, 202 first-time adolescent mothers (12–19 years) had been identified. Of this group, 10 (4.9%) could not be located and so did not complete the PGS interview in the year following delivery (T2). Of the remaining 192 participants, we excluded 33 mothers with PGS interviews that were completed more than 6 months following childbirth to retain a focus on the highest risk period for postpartum psychopathology [39, 54]. We ran attrition analysis to determine whether the 43 missing or excluded individuals differed from the retained mothers on the T1 variables (described below). The results showed no differences with the exception of maternal age: the excluded/missing adolescents were older, with a mean age of 17.14 years (SD = 1.54), compared with a T1 mean age of 15.25 years (SD = 1.38) in the included group (F [1,188] = 58.84, *p* < .001).

Psychosocial risk factors were assessed in the PGS wave that occurred immediately prior to each childbearing adolescent’s pregnancy. The timing of this pre-pregnancy assessment (denoted by T1) was determined by subtracting 42 weeks from each adolescent’s date of delivery and using data from the PGS interview that occurred immediately prior to this date. Based on this criterion three childbearing adolescents were excluded from the analysis because they had not completed the annual PGS interview occurring at the T1 assessment wave. As described below, risk-set propensity scores were then used to identify non-childbearing adolescents matched at T1 from the remaining pool of 2,303 PGS participants.

Approval for all study procedures was obtained from the University of Pittsburgh Human Research Protection Office. Written informed consent was obtained prior to data collection. For participants aged 17 and younger, written consent was obtained from the caregiver and verbal assent was obtained from the adolescent. From age 18 onwards, participants provided their own written consent. In-person interview data were collected in the home on an annual basis by trained PGS interviewers using laptop computers. All the participants were financially reimbursed for their help with the research.

### Measures

All measures were administered at T1, with assessments of depression, conduct problems, and substance use also conducted at T2 to evaluate change.

Demographic data were collected via caregiver report and included information on the adolescent’s age, race (coded as 0 = European American, 1 = African American), and household composition (0 = living in a dual-parent household, 1 = living with a single parent).

Severity of depression and conduct problems were assessed using the DSM-IV based Adolescent Symptom Inventory-4 (ASI-4) [55]. Adolescents reported on the presence of nine DSM-IV symptoms of major depressive disorder [56] plus two additional symptoms: low self-esteem and hopelessness. Seven of the symptoms are rated on four-point scales (0 = never to 3 = very often), and four symptoms (significant change in normal appetite or weight, sleep, activity and concentration) are scored as 0.5 = absent or 2.5 = present. Changes in appetite/weight assessed at T2 were omitted from the current analyses due to the likely confound with postpartum status [57]. In addition, the sleep disturbance symptom was administered with the clarification that it was not related to getting up in the night to attend to the baby. Symptom ratings were summed to generate a depression severity score. Whereas all 11 depression symptoms contributed to the risk-set propensity score described below, 10 symptoms (excluding the appetite/weight) were used to examine T1-T2 change in depression severity in order to ensure comparable measurement across the childbearing and non-childbearing groups.

Severity of conduct problems was assessed using caregiver and adolescent reports of DSM-IV symptoms of conduct disorder [56]. All 15 items were rated on four-point scales (0 = never to 3 = very often). Following prior work [58, 59], a best-estimate approach was taken in which the highest rating between the two informants was used for each item and items were then summed to create a conduct problem severity score. The ASI-4 has shown adequate concurrent validity, and sensitivity and specificity of depression and conduct disorder symptom scores to clinicians’ diagnoses [55]. In the present study, internal consistency at the sample mean age was α = .83 for depression severity (both with and without the symptom assessing change in appetite/weight) and α = .70 for conduct problems.

Substance use was assessed at T1 and T2 using adolescent reports on the Nicotine, Alcohol and Drug Use scale [60]. Three items assessed frequency of tobacco, alcohol and marijuana use in the past year on 8-point rating scales (0 = none, 1 = less than 5 times, 2 = more than 5 times but less than once a month, 3 = about once a month, 4 = about once a week, 5 = a couple of times a week, 6 = nearly every day, 7 = every day or more than once a day).

Low parental warmth was assessed by caregiver report using six items of the Parent-Child Rating Scale [61] at T1. Items (e.g. ‘How often have you wished she would just leave you alone’) were scored on 3-point scales (1 = almost never to 3 = often). Internal consistency was good, indicated by Cronbach’s *α* = .72. Harsh punishment was assessed by combining five items of psychological aggression and one item on spanking from the Conflict Tactics Scale: Parent-child version [62]. Adolescents responded to items (e.g. ‘In the past year, if you did something that you are not allowed to do or something that [your caregiver] didn’t like, how often did s/he shout, yell, or scream at you’) using a 3-point answer format (1 = never to 3 = often). Good discriminant and construct validity have been reported for this measure [62]. In the present sample, the internal consistency coefficient was α = .73.

Sexual abuse was assessed at T1 from the combined reports of the caregiver and adolescent. Caregivers were asked whether someone had made his/her child see or do something sexual that she didn’t want to see or do, like touching in a sexual way, exposing self or masturbating, or engaging in sexual intercourse (0 = no, 1 = yes). The adolescent was also asked four yes/no questions related to being touched or having pictures taken of private parts in an unwanted way, being made to touch someone else’s private body parts or made to watch other people having sex or doing things with their private parts when she didn’t want to. Endorsement of the caregiver item or any adolescent item was coded as experienced sexual abuse.

Peer Victimization at T1 was assessed by adolescent report using the Peer Experiences Scale [63]. Adolescents responded to nine items assessing frequency of victimization during the past three months (e.g. ‘A student hit, kicked or pushed me in a mean way’) on 5-point rating scales (0 = never to 4 = a few times a week). Good reliability and concurrent validity have been reported for the scale [63], with high internal consistency (α = .86) in the current sample. Affiliation with deviant peers was assessed using adolescent report on the 11-item Peer Delinquency Scale [61]. This measure assesses the number of deviant behaviors engaged in by one or more peers. Items (e.g. ‘Has/have your friend(s) used, or threatened to use, physical force in order to be the boss’) were scored as 0 = no or 1 = yes for girls reporting a single friend, and ranging from 0 = none to 3 = all of them, for girls with multiple close friends. For the current analyses, the response format for girls reporting more than one close friend was collapsed to a binary response indicating whether none (0) vs. one or more peers (1) engaged in each behavior. Endorsed items were then summed to create a total score ranging from 0–11. The internal consistency of these items was α = .80.

Social adversity was assessed using caregiver report on the 28-item Difficult Life Circumstances measure [64]. Items (e.g. ‘Do you get hassled pretty often by bill collectors or collection agencies?’) are scored with a no (0) yes (1) format. The measure has good psychometric properties [64]; in the current sample Cronbach’s α was .69. Neighborhood problems were assessed with the Your Neighborhood questionnaire [61]. This 17-item measure lists potential problems such as vandalism, burglaries and drug use scored on 3-point scales (1 = not a problem to 3 = a big problem), and has excellent internal consistency (α = .96).

### Risk-set propensity score matching procedure

We used risk-set propensity score matching [65] to identify a group of non-childbearing adolescents similar to childbearing adolescents at T1. Risk-set propensity score matching is a method used to study the effects of time-varying ‘treatments’, in this case girls becoming pregnant at different ages during adolescence. The aim in using this method was to select non-childbearing adolescents to form a comparison group with a similar distribution of covariates as childbearing mothers in the assessment period immediately prior to pregnancy. Risk-set propensity scores used in the matching process were derived from 15 variables assessed at T1 (Table 1). A ‘nearest neighbor without replacement’ method was used to select two non-childbearing controls for each childbearing individual with similar risk-set propensity scores in the respective assessment period. The minimum matching requirement was that their propensity score was within a ‘caliper’ of .05 of the childbearing adolescent. The distribution of propensity scores in the childbearing and non-childbearing PGS participants showed substantial overlap for the matching procedure. The success of the propensity score matching depends on a) how many childbearing adolescents can be matched, and b) the ‘balance’ of the matched non-childbearing adolescents on covariates (see below). Of the 156 childbearing adolescents with risk-set propensity scores, the remaining pool of 2,303 PGS participants enabled at least two matched non-childbearing controls to be identified for 147 adolescents, a single matched control for a further six, and no matches with a propensity score within the .05 caliper could be found for three childbearing adolescents. To optimize statistical power and reduce standard errors [66, 67], we elected to use the 147 childbearing adolescents with two matched controls (n = 294) in the current analyses.

**Table 1:** Descriptive statistics of childbearing and matched non-childbearing adolescents.

	Childbearing adolescents (n = 147)			Non-childbearing matches (n = 294)			
Time 1	Mean (SD)	range	N (%)	Mean (SD)	range	N (%)	*d*
Adolescent age	15.25 (1.38)	12–17		15.25 (1.39)	12–18		.10
African American race			126 (85.7)			264 (89.8)	-.11
Living with a single parent			98 (66.7)			208 (70.7)	-.09
Depression ^a^	7.56 (5.22)	2–26		7.76 (5.28)	2–28		-.03
Conduct problems	2.36 (2.67)	0–12		2.18 (2.76)	0–16		.04
Tobacco use	1.16 (2.44)	0–7		.87 (2.03)	0–7		.13
Alcohol use	.54 (1.07)	0–5		.56 (1.13)	0–6		-.02
Marijuana use	1.01 (2.04)	0–7		.81 (1.73)	0–7		.11
Low parental warmth	9.10 (2.42)	6–16		8.85 (2.38)	6–18		.10
Harsh punishment	9.18 (2.62)	6–17		9.13 (2.50)	6–17		.02
Sexual abuse			6 (4.1)			8 (2.7)	-.03
Peer victimization	2.63 (3.80)	0–25		2.43 (3.47)	0–23		.05
Deviant peers	5.60 (3.23)	0–11		5.65 (3.20)	0–11		-.01
Social adversity	3.49 (2.95)	0–12		3.59 (2.63)	0–14		-.04
Neighborhood problems	25.22 (8.04)	17–48		25.22 (9.05)	17–51		-.12
						***F (1*,*439)*** ^b^	***d***
T1 to delivery gap (months)	15.72 (4.99)	10–28					
Time 2 adolescent age	16.97 (1.45)	13–19		16.97 (1.45)	13–20	.16	0
Delivery to T2 gap (months)	3.72 (1.47)	1–6					
T1 to T2 gap (months)	19.34 (5.14)	10–34		19.13 (5.33)	11–32	0	0

Notes

^a^ Depression severity score and diagnosis excludes DSM-IV symptom assessing change in weight/appetite

^b^ Neither of the F statistics indicated significant group differences (*p* < .05); *d* = Cohen’s d effect size.

The childbearing adolescents who either could not be matched (n = 3) or could only be matched with one non-childbearing adolescent (n = 6) differed from the 147 childbearing adolescents matched with two controls on three variables. These nine adolescents had more severe conduct problems (Mann-Whitney *z* = -.20, *p* < .05), and were more likely to be using tobacco (*X*^*2*^[1] = 14.19, *p* < .001) and marijuana (*X*^*2*^[1] = 6.98, *p* < .01) at T1. There were no differences however, between the included and excluded childbearing adolescents on demographic variables (T1 or T2 age, race, living with a single parent), depression severity, experienced sexual abuse, parenting or peer factors, social adversity or neighborhood problems.

Balance was determined by comparing covariates between matched childbearing and non-childbearing adolescents, and calculating the standardized mean differences between them. The conventional threshold for a small standardized mean difference is *d* < .20 [68]. A comparison of the childbearing adolescents and the matched non-childbearing adolescents on variables used to form the propensity scores, showed that all *d* values were less than 0.13 (Table 1), indicating that the matching process was successful. This step is critical to establish that any subsequent group differences were not a function of residual confounding between adolescent pregnancy risk and postpartum adjustment.

### Data Analysis

All data were analyzed in IBM SPSS 22.0 [69]. Square root transformations were applied to the depression and conduct problems severity scores and frequency of substance use to reduce moderate positive skew [70].

We tested our first hypotheses examining T1 to T2 changes in the severity of depression and conduct problems symptoms, and frequency of substance use among childbearing mothers using paired samples t-tests. Our second set of hypotheses was tested using separate independent samples t-tests. In these analyses, T1 to T2 change scores in symptom severity, and frequency of substance use were compared between the childbearing and matched non-childbearing groups. Given that change was computed from T2-T1, a negative mean difference indicated a decrease in severity, frequency or level over time. Because the paired t-test is a correlated design with the potential to artificially inflate effect sizes [71], the original means and standard deviations rather than the paired *t*-test values were used to compute Cohen’s *d*. Cohen’s *d* of .20 was considered a small effect, .50 a medium effect and .80 a large effect [72].

## Results

Descriptive statistics for the sample are shown in Table 1. The mean age of the childbearing adolescents at time T1 was 15.25 years (SD = 1.38), 86% were African American and 67% were living with a single parent. The mean depression and conduct problems scores fell in the moderate severity range [55]. All the *d* values for T1 variables were within the bounds of ± .20, indicating that the matching procedure was successful. The mean timing of the T1 interview was 15.72 months (SD = 4.99) prior to delivery, and the postpartum assessment (T2) occurred when the infant was between 1 and 6 months of age (mean timing = 3.72 months, SD = 1.47). There were no group differences in adolescent age at T2, nor in the duration of the T1 to T2 interval (*η*^2^ < 0.01, indicating a small effect size [72]).

Paired t-tests were conducted to examine change in symptom severity, and frequency of substance use between T1 and T2 among the childbearing adolescents. The results showed no significant within-subject change in the severity of depressive symptoms from pre-pregnancy to the postpartum period (Table 2). In contrast, the severity of conduct problems showed a significant reduction across this interval (mean difference = -.46, SD = .98; *t* = 5.73, *df* = 146, *p* < .001, *d* = .52). Adolescent reports of frequency of marijuana use also decreased from pre-pregnancy to postpartum (mean difference = -.15, SD = .83; *t* = 2.26, *df* = 146, *p* < .05, *d* = .19). Finally, the results indicated that there was no change in the frequency of adolescents’ reported use of tobacco or alcohol within the childbearing group across the transition to motherhood.

**Table 2:** Change in symptom severity and frequency or substance use between T1 and T2 within the childbearing group (N = 147).

	T2-T1 Mean difference (SD)	*t* (146)	*95% CI*	*d*
Depression ^a^	-.14 (1.06)	1.56	-1.56–.04	.13
Conduct problems ^a^	-.46 (.98)	5.73***	-.62–-.30	.52
Tobacco use ^a^	.03 (.76)	.51	-.09–.16	.03
Alcohol use ^a^	.03 (.83)	.43	-.11–.16	.04
Marijuana use ^a^	-.15 (.83)	2.26*	-.29–-.02	.19

Notes

^a^ T2-T1 mean difference reflects difference in square-root transformed data. Negative mean difference values indicate a decrease in level over time; *d* = Cohen’s *d* effect size

***p < .001

*p < .05

**Fig 1:**
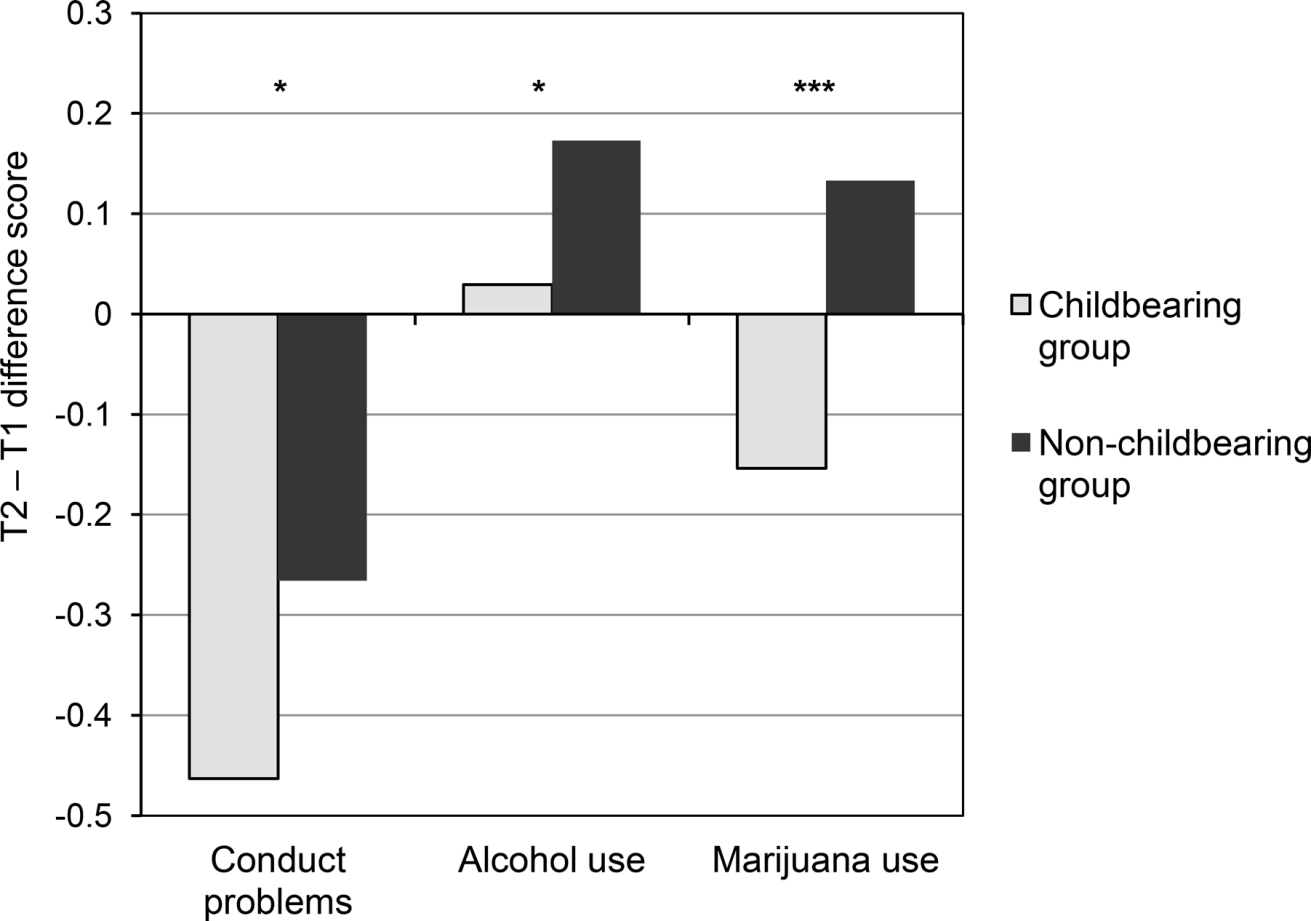
Change in conduct problems and substance use between T1 and T2 by childbearing status. Notes: T2-T1 mean difference reflects difference in square-root transformed data; * *p* < .05; ***p < .001.

The extent of changes in T1 to T2 depression and problem behaviors among the childbearing adolescents (n = 147) were then examined relative to the matched non-childbearing control group (n = 294). The results showed no group difference in reported depression severity scores between T1 and T2, indicating no effect of childbearing status on change (or lack thereof) in depressed mood (Table 3). In contrast, a group difference was revealed for change in conduct problems severity. While there was an overall decrease in the severity of conduct problems for both groups, the reduction among childbearing adolescents was greater than their matched controls (*t* = 2.06, *df* = 439, *p* < .05, *d* = .20). This pattern is illustrated in Fig 1. For substance use, the results showed a significant effect of childbearing status on change in the frequency of marijuana use across the interval from T1 to T2 (*t* = 3.38, *df* = 439, *p* < .01, *d* = .32, a small-to-moderate effect size), and a marginal group difference for change in alcohol use (*t* = 1.95, *df* = 439, *p* = .05, *d* = .19). The childbearing group showed a mean reduction in marijuana use, whereas the matched non-childbearing group reported a mean increase (Fig 1). In addition, compared with the lack of change in frequency of alcohol use reported by the childbearing adolescents across the T1 to T2 period noted previously, the matched non-childbearing controls showed a mean increase in the frequency of alcohol use across the same period. No group differences were revealed for change in tobacco use frequency.

**Table 3:** Change in symptom severity and frequency or substance use between T1 and T2 by childbearing and non-childbearing groups.

	Childbearing (N = 147)	Non-childbearing (N = 294)			
	T2-T1 Mean difference (SD)	T2-T1 Mean difference (SD)	*t* (439)	*95% CI*	*d*
Depression ^a^	-.14 (1.06)	-.17 (.97)	.28	-.17–.23	.03
Conduct problems ^a^	-.46 (.98)	-.27 (.93)	2.06*	-.38–-.01	.20
Tobacco use ^a^	.03 (.76)	.14 (.79)	1.35	-.26–.05	.13
Alcohol use ^a^	.03 (.83)	.17 (.68)	1.95*	-.29–-.01	.19
Marijuana use ^a^	-.15 (.83)	.13 (.85)	3.38**	-.45–-.12	.32

Notes

^a^ T2-T1 mean difference reflects difference in square-root transformed data. Negative mean difference values indicate a decrease in level over time; *d* = Cohen’s *d* effect size

**p < .001

*p ≤. 05

## Discussion

Results from the current study contribute to knowledge on adolescent postpartum adjustment in several unique ways. The study used a risk-set propensity score matching procedure to enable a rigorous examination of postpartum risks for depression, conduct problems and substance use that were distinct from selection effects associated with becoming pregnant in adolescence. To achieve this, the study utilized prospectively gathered, repeated measures data from a representative population-based study with low attrition that began in childhood. By leveraging the resources of this large dataset, it was possible to identify propensity-matched non-childbearing adolescents who were also matched on the length of follow-up interval, in order to also rule out potential effects of normative developmental change on postpartum adjustment.

The findings from the study provide no evidence that adolescents are at elevated risk for depression in the postpartum period. Thus, within the childbearing group, adolescents reported no change in depression severity across the interval from pre-pregnancy to postpartum. This lack of change between T1 and T2 was also demonstrated among matched non-childbearing controls. Taken together, these results indicated that the experience of becoming a mother had neither a positive nor a negative effect on depression vulnerability regardless of propensity to become pregnant during adolescence or normative patterns of development. Furthermore, because the mean depression severity scores in the current study fell within the ‘moderate’ range according to ASI-4 normative data [55] and adolescents’ reports included a full range of scores, these null results are unlikely to be a function of floor effects or low variability in depressive symptoms in the current sample. Although we expected an increase in depression severity among the non-childbearing adolescents between T1 and T2, it is possible that the interval (mean = 19.3 months) was too short to capture increasing trends in depression that occur over the span of adolescence [31, 33]. Instead, the results are consistent with reports of depression stability in community-based samples of non-childbearing females [73], and for the childbearing adoelscents specifically, the findings concur with the retrospective reports of adult women indicating that pre-existing depressive symptoms often continue into the postpartum period [28, 29].

Our hypothesis that the severity of conduct problems among childbearing adolescents would reduce across the interval spanning childbirth was supported in the current study. The results also showed that this reduction was greater among the childbearing adolescents than their age- and propensity-matched controls, suggesting an effect that was specific to becoming an adolescent mother. This findings is consistent with the notion that childbearing limits opportunities to engage in aggressive, destructive or deceitful behaviors outside the home [42], as well as the possibility that childbearing confers a shift in roles and future expectancies that have a positive or ‘correcting’ effect on the lives of adolescents [45, 74]. However, further work is needed to elucidate these mechanisms and determine how they might affect future functioning such as the capacity to provide sensitive parenting to offspring.

Consistent with prior reports showing a variety of patterns of adolescent substance use across the transition to motherhood [40, 41], change patterns in the current study were found to differ by substance type. Thus for marijuana, we observed a robust decrease in use from pregnancy to the postpartum period among childbearing adolescents that contrasted with increasing frequency of use among their matched controls as expected during this developmental period [75]. This decrease among childbearing adolescents is notable given the relatively low frequency of use reported by the sample as a whole at T1. The finding suggests that adolescent childbearing status is directly linked to reduced marijuana use, and furthermore runs counter to the typical increasing patterns of adolescent marijuana use. When frequency of alcohol use among childbearing adolescents at T1 and T2 was examined, the results revealed high levels of stability and minimal change over time. This pattern of no change also contrasted with increasing rates of use among matched controls, although the effect size was small. Nevertheless, these findings indicate a relative reduction in alcohol use among childbearing compared with matched controls. These actual and relative reductions in marijuana and alcohol use are encouraging in terms of reducing risks to the offspring either from fetal exposure or compromised parenting, and they reinforce the notion that the peripartum period is an important window of opportunity to encourage maintenance of healthy behaviors [76]. However, it will be important to determine whether these effects are sustained over time given reports that reductions in adolescent substance use often reverse over the years following childbirth [37]. Finally, the current study findings revealed no change in the frequency of tobacco use from pre-pregnancy to the postpartum period in either the childbearing or the non-childbearing groups; a pattern that may reflect the greater addictive properties of nicotine relative to other substances [77].

The findings presented here should be considered in the context of several limitations. First, the measure used to assess depression severity has not been validated for use in the postpartum period. A major challenge for prospective research examining continuities and discontinuities in psychopathology across the peripartum period is that the assessment of somatic symptoms of depression is likely to be confounded with pregnancy or postpartum status [57]. In an attempt to address this issue but enable the same age-appropriate measure to be used at both time points, and with both childbearing and non-childbearing adolescents, we omitted one symptom assessing change in appetite/weight from all analyses. We also sought evidence for construct validity of the measure with an established postpartum screening measure, which has been validated on both adults and adolescents [78, 79]. The moderate correlation between the scores of the two questionnaires helped to allay concerns about measurement validity. Second, the current study utilized two data points, which prevented examination of trajectories of the dependent variables across pregnancy and into the postpartum period. Although many childbearing adolescents were interviewed during pregnancy as part of the annual PGS data collection schedule, prenatal data were unavailable for a proportion of participants. As a result, we elected to focus on change from pre-pregnancy to postpartum when more complete data could be obtained. It is possible, however, that the developmental course of depression and problem behaviors differed by childbearing status in unmeasured but important ways. Third, the large-scale multi-wave design of the PGS meant that it was not feasible to collect biological measures (e.g. urine, hair) to corroborate adolescent reports of substance use. It is therefore possible that social stigma or fear of negative consequences may have led the childbearing adolescents to under-report their use of substances in the postpartum period. Concerns of differential reporting bias are reduced however, by the lack of a group difference in reported tobacco use, given that cigarette smoking in the peripartum period has received more public health attention as a teratogen than marijuana [80]. In addition, it is possible that our measures of substance use did not capture rebound effects among the few participants assessed within the first postpartum months. Finally, although every effort was made to obtain a representative sample of young mothers, several sources of missing data should be noted. To begin, a small percentage of adolescents were omitted from the current analyses due to missing PGS data at either T1 (1.9%) or at T2 (4.9%). Because we elected to focus on the highest risk period for postpartum psychopathology [39, 54], 33 childbearing adolescents (17.2%) whose T2 data occurred beyond 6 months postpartum were excluded. Although attrition analyses indicated that the retained childbearing adolescents were significantly younger, they did not appear to differ in any other substantive way. Lastly, for the sake of statistical power [66], we chose to retain childbearing adolescents for whom we could identify two propensity matched controls, with the result that nine more-difficult-to-match childbearing adolescents were excluded. This excluded group was characterized by high levels of conduct problems and tobacco and marijuana use, but did not differ from the retained group on any other propensity variables such as depression severity, deviant peers and neighborhood disadvantage. These decisions were made with the goal of increasing specificity and statistical power, but it is acknowledged that our approach may have reduced the generalizability of the results for particular subgroups of childbearing adolescents (e.g., older adolescents, adolescents with more conduct problems and more frequent use of tobacco and marijuana).

In summary, the current study provided no support for the notion that adolescent childbearing is associated with vulnerability for depression, conduct problems or substance use. Although being born to an adolescent mother is associated with increased risk for mental health problems in offspring across their lifespan [25, 81], the current study suggests that postpartum depression and problem behaviors may not add to these risks. Nevertheless, further research is clearly warranted to determine whether the findings reported here are robust over a longer period, and to begin to investigate which aspects of childbearing (e.g. hormonal exposure, shift from self- to other-focus) might protect or increase risk for particular groups of adolescents.
